# Percutaneous needle biopsy for indeterminate renal masses: a national survey of UK consultant urologists

**DOI:** 10.1186/1471-2490-7-10

**Published:** 2007-07-04

**Authors:** Azhar A Khan, Iqbal S Shergill, Sheila Quereshi, Manit Arya, Mohammed T Vandal, Sandeep S Gujral

**Affiliations:** 1 Bristol Urological Institute, Southmead Hospital, Bristol, UK; 2 Department of Urology, Harold Wood Hospital, Essex, UK

## Abstract

**Background:**

The use of percutaneous needle biopsy in the evaluation of indeterminate renal masses is controversial and its role in management remains largely unclear. We set to establish current practice on this issue in UK urology departments.

**Methods:**

We conducted a national questionnaire survey of all consultant urologists in the UK, to establish current practice and attitudes towards percutaneous needle biopsy in the management of indeterminate renal masses.

**Results:**

139 (43%) consultant urologists never use biopsy, whereas 111 (34%) always employ it for the diagnosis of indeterminate renal masses. 75 (23%) urologists use biopsy only for a selected patient group. Mass in a solitary kidney, bilateral renal masses and a past history of non-renal cancer were the main indications for use of percutaneous biopsy. The risk of false negative results and biopsy not changing the eventual management of their patients were the commonest reasons not to perform biopsy.

**Conclusion:**

There is a wide and varied practice amongst UK Consultant Urologists in the use of percutaneous biopsy as part of the management of indeterminate renal masses. The majority of urologists believe biopsy confers no benefit. However there is a need to clarify this issue in the wake of recent published evidence as biopsy results may provide critical information for patients with renal masses in a significant majority. It not only differentiates benign from malignant tissue but can also help in deciding the management option for patients undergoing minimally invasive treatments.

## Background

Renal masses represent a variety of benign and malignant neoplasms and generally the diagnosis is based on findings at cross sectional imaging [1]. Advancement in the resolution of diagnostic radiological modalities especially computed tomography (CT) has not only led to better characterization of these lesions but also caused a dramatic rise in the incidentally discovered renal masses. Currently, more than one third of renal tumours are discovered incidentally [2]. Despite these advances, it is not uncommon to see radiologists labeling a renal mass as indeterminate in contemporary practice, especially small lesions [3]. Management of this subgroup of patients, in whom imaging is inconclusive, poses diagnostic and therapeutic dilemmas. In addition, recently, minimally invasive ablative methods such as radio frequency ablation (RFA) and cryotherapy have shown great promise in the treatment of small renal masses and it is well understood that biopsy may provide the only chance to get a tissue diagnosis in such cases. As there is controversy in the use of needle biopsy in the evaluation of indeterminate renal masses, we aimed to survey current practice in the United Kingdom, amongst consultant urologists regarding this issue.

## Methods

**Figure 1:**
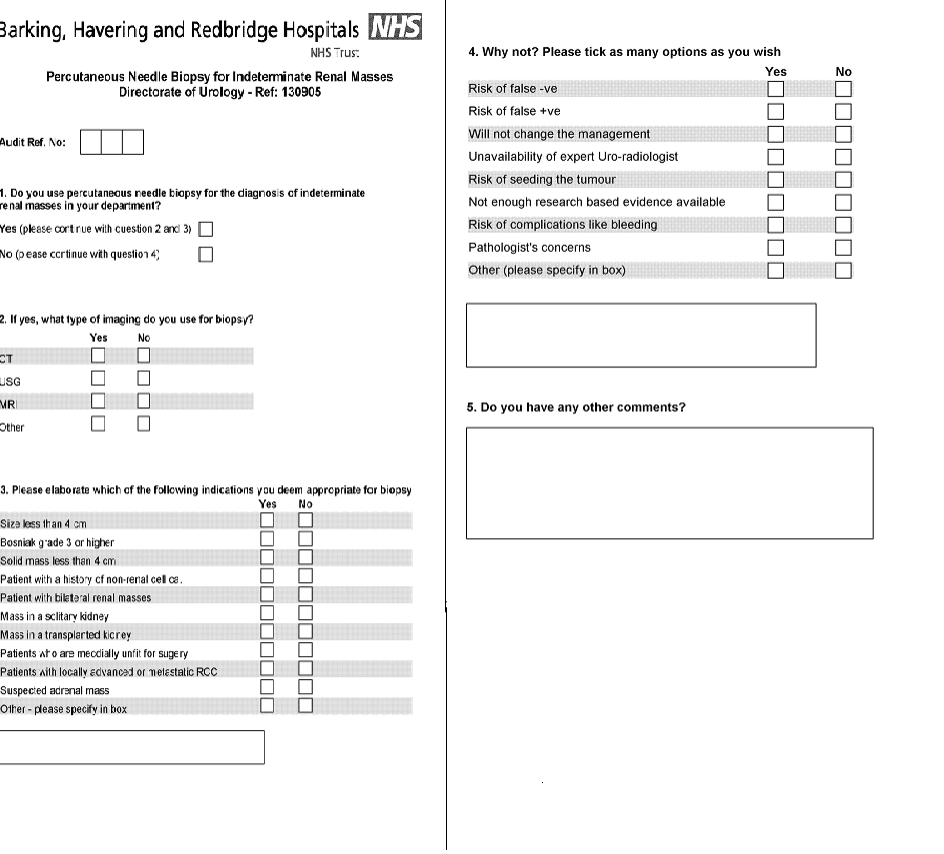
Questionnaire.

A standardised questionnaire was sent to all UK consultant urologists on the British Association of Urological Surgeons (BAUS) register in October 2005. The questionnaire aimed to highlight individual practice regarding the use of percutaneous needle biopsy in the management of indeterminate renal masses (Figure 1).

The participants were asked initially whether they used needle biopsy in their practice and, if so, the indications for its use along with their preferred imaging modality employed to direct biopsy. If biopsy was not used, factors precluding its use were established. Finally, perceptions, in the form of general comments were also requested. Recipients were asked to return the questionnaire in the self-addressed, stamped reply envelope.

## Results

Of the 525 questionnaires sent out, 336 (64%) were returned, of which 11 were excluded because of incomplete answers. Hence, a total of 325 responses were therefore analysed.

139 (43%) consultant urologists never use biopsy, whereas 111 (34%) always employ it for the diagnosis of indeterminate renal masses. Moreover, 75 (23%) urologists answered both yes and no to the first question on the questionnaire and mentioned that they use biopsy only for a selected patient group. As shown in Table 1, mass in the solitary kidney (57%), bilateral renal masses (51%), and past history of non-renal cancer (46%) are the main indications used by the participants who employ percutaneous biopsy in their practice. Medically unfit patients with a renal mass, multidisciplinary team (MDT) decision after inconclusive radiology and patients with possible metastatic RCC are less common indications. Though not asked in the questionnaire as a separate indication, 39 urologists described lymphoma as an indication.

**Table 1:** Indications for biopsy

**Indications**	%
Mass solitary kidney	57%
Bilateral renal masses	51%
History of non-renal cancer	43%
Medically unfit	36%
MDT decision	35%
Metastatic RCC	29%
Lymphoma	21%

Of the participants who do not use biopsy, 87% described false negative results as the main reason, whereas 58% believe that use of biopsy would not change the eventual management of their patients (Table 2). Tumour seeding, biopsy related complications, histopathological concerns, false positive results and lack of available uro-radiologist were other less common factors mentioned against the biopsy.

**Table 2:** Reasons for not choosing to perform a biopsy

**Reasons**	**%**
False -ve results	87%
Will not alter management	58%
Tumour seeding	42%
Complications	27%
Pathologist's concern	24%
False +ve results	15%
Unavailability of uro-radiologists	6%

## Discussion

There is a great deal of controversy in the management of patients found to have indeterminate renal masses [1,2,4]. Traditionally patients are either offered radical surgical procedures or active surveillance, even though it is well known that 20% of patients undergoing operation for a suspicious renal mass will have a histopathologically benign lesion [5].

Percutaneous needle biopsy has been an accepted diagnostic tool for solid intraabdominal masses, but its role has largely remained unclear in the evaluation of solid renal tumours. Our survey shows that currently only 34% urologists use biopsy in their practice for renal tumours. Biopsy can provide a definitive tissue diagnosis to direct future therapy in patients with inoperable disease because of locally advanced RCC and the presence of metastatic disease and comorbidities [6]. Indications for biopsy in contemporary urology though are expanding rapidly.

Some of the suggested indications in the recent literature include small solid renal masses that do not fit the radiological features of typical RCC and can form up to 50% of suspicious renal masses [7]. Biopsy can be used to avoid unnecessary surgery in these patients. On the other hand, minimally invasive therapies such as Radio frequency ablation (RFA) and cryotherapy are increasingly employed in the management of small renal tumours especially less than 4 cm [8]. Pre-operative percutaneous needle biopsy may be the only opportunity to obtain a tissue diagnosis [9].

This survey showed that the majority of UK urologists do not use needle biopsy or use it only under special circumstances (66%). Their major concern is high false negative results and especially the problem of managing a negative biopsy. They also suggested that biopsy will not alter the management of their patients. Although these concerns are valid, many recent studies have shown that biopsy may significantly alter the management of indeterminate renal masses. Wood et al reported only 6% false negative results in 79 biopsies and Neuzillet et al reported a false negative result of 5.6% in 88 biopsies [10,11]. Not surprisingly most of these false negative results were because of insufficient tissue material. Clinical management was altered due to biopsy in more than 40% of patients in both studies. Bosniak classification for complex cysts is not reliable and can lead to mismanagement [12]. Richter et al assigned a definite diagnosis to 89.4% of patients with Bosniak II and III cysts on the basis of CT guided biopsy [13].

In addition, 24% of consultant urologists think that pathologists have concerns against the use of this technique for categorisation of renal tumours into clinically relevant histological categories that can influence the management decisions. With the possibility of inconclusive cytology, the diagnostic yield of aspiration cytology in the evaluation of renal tumours is not considered to be reliable [14]. Results can be improved by using core biopsy in preference to fine needle aspiration cytology [15] or a combination of both techniques [10]. This can provide better architectural information about the tissue. This technique can also provide tissue for additional histopathological and biochemical procedures. Lactate dehydrogenase (LDH) and protein assessment of the biopsy specimens can be used to differentiate neoplastic from inflammatory lesions [13]. In some cases distinction between chromophobe RCC, oncocytoma and even clear cell RCC (eosinophilic variant) can be problematic. [16] Adult papillary renal tumors with oncocytic cells might be a distinct variant in the papillary renal cell carcinoma group. [17] Shah et al advised the use of Hale's colloidal iron and contemporary immunohistochemical panel in all such cases to define morphology [9]. According to them all equivocal cases should undergo excision. By adopting these strategies pathologists can play an important role in the initial diagnosis and subsequent management of indeterminate renal tumours.

In this survey, 42% of participants were concerned about needle track seeding of tumour cells. This risk is increased in patients with transitional cell carcinoma and probably is more frequent with non-cutting needles than with cutting needles [18]. In addition, most recent studies have reported no such complication even after long follow-up [10,11,13-15]. Richter et al have attributed this to the use of a coaxial system, which shields the tissue outside Gerota's fascia. [13]

Another concern regarding percutaneous biopsies has been the potential for other complications such as bleeding and haematoma (27%). This risk appears to be small with fine needles than larger needles [19], and will only require conservative management in the majority of cases. Results can also be improved by performing biopsies only in highly specialised uro-radiology centres by expert radiologists. Helical CT guidance should be used in preference to other modalities as it allows accuracy and fast image acquisition [11].

## Conclusion

Our survey shows a wide and varied practice amongst UK Consultant Urologists in the management of indeterminate renal masses and much of it is not evidence based. The majority of urologists believe biopsy confers no benefit. However there is a need to clarify this issue in the wake of recent published evidence. Biopsy results may provide critical information for patients with renal masses in a significant majority. It not only differentiates benign from malignant tissue but can also help in deciding the management option for patients undergoing minimally invasive treatments.

## Competing interests

The author(s) declare that they have no competing interests.

## Authors' contributions

Azhar Khan designed the questionnaire, conducted the survey, analysed the data and drafted the manuscript. Iqbal Shergill and Sheila Quereshi made substantial contribution to the collection of the data and assisted in the preparation of manuscript. Manit Arya made substantial contribution to the design of the study and revised the manuscript. M Tanvir Vandal and Sandeep Gujral conceived the study, helped in the design of the questionnaire and finalized the manuscript. All authors read and approved the final manuscript.

## Pre-publication history

The pre-publication history for this paper can be accessed here:



## References

[B1] Wolf JS (1998). Evaluation and management of solid and cystic renal masses. J Urol.

[B2] Rodriguez R, Fishman EK, Marshall FF (1995). Differential Diagnosis and Evaluation of the Incidentally Discovered Renal Mass. Seminars in Urological Oncology.

[B3] Smith PS, Marshall FF, Fishman EK (1998). Spiral computed tomography evaluation of the kidneys: state of the art. Urology.

[B4] McCredie M (1994). Bladder and kidney cancers. Cancer Surv.

[B5] McKiernan J, Yossepowitch O, Kattan MW, Simmons R, Motzer RJ, Reuter VE, Russo P (2002). Partial nephrectomy for renal cortical tumors: pathological findings and impact on outcome. Urology.

[B6] Niceforo JR, Coughlin BF (1993). Diagnosis of renal cell carcinoma: value of fine-needle aspiration cytology in patients with metastases or contraindications to nephrectomy. AJR.

[B7] Lechevallier E, Andre M, Barriol D, Daniel L, Eghazarian C, De Fromont M, Rossi D, Coulange C (2000). Fine-needle percutaneous biopsy of renal masses with helical CT guidance. Radiology.

[B8] Derweesh IH, Novick AC (2003). Small renal tumours: natural history, observation strategies and emergency modalities of energy based tumor ablation. Can J Urol.

[B9] Shah RB, Bakshi N, Hafez KS, Wood DP, Kunju LP (2005). Image-guided biopsy in the evaluation of renal mass lesions in contemporary urological practice: indications, adequacy, clinical impact and limitations of the pathological diagnosis. Human Pathology.

[B10] Wood BJ, Khan MA, McGovern F, Harisinghani M, Hahn PF, Mueller PR (1999). Imaging guided biopsy of renal masses: indications, accuracy and impact on clinical management. J Urol.

[B11] Neuzillet Y, Lechevallier E, Andre M, Daniel L, Coulange C (2004). Accuracy and clinical role of fine needle percutaneous biopsy with computerized tomography guidance of small (less than 4.0 cm) renal masses. J Urol.

[B12] Wilson TE, Doelle EA, Cohan RH, Wojno K, Korobkin M (1996). Cystic renal masses: a reevaluation of the usefulness of the Bosniak classification system. Acad Radiol.

[B13] Richter F, Kasabian NG, Irwin RJ, Watson RA, Lang EK (2000). Accuracy of diagnosis by guided biopsy of renal mass lesions classified indeterminate by imaging studies. Urology.

[B14] Campbell SC, Novick AC, Herts B, Fischler DF, Meyer J, Levin HS, Chen RN (1997). Prospective evaluation of fine needle aspiration of small, solid renal masses: accuracy and morbidity. Urology.

[B15] Vasudevan A, Davies RJ, Shannon BA, Cohen RJ (2006). Incidental renal tumours: the frequency of benign lesions and the role of preoperative core biopsy. BJUI.

[B16] Abrahams NA, MacLennan GT, Khoury JD, Ormsby AH, Tamboli P, Doglioni C, Schumacher B, Tickoo SK (2004). Chromophobe renal cell carcinoma: a comparative study of histological, immunohistochemical and ultra structural features using high throughput tissue microarray. Histopathology.

[B17] Lefevre M, Couturier J, Sibony M, Bazille C, Boyer K, Callard P, Vieillefond A, Allory Y (2005). Adult papillary renal tumor with oncocytic cells: clinicopathologic, immunohistochemical, and cytogenetic features of 10 cases. Am J Surg Pathol.

[B18] Herts BR, Baker ME (1995). The current role of percutaneous biopsy in the evaluation of renal masses. Semin Urol Oncol.

[B19] Cozens NJ, Murchison JT, Allan PL (1992). Conventional 15 G needle technique for renal biopsy compared with ultrasound-guided springloaded 18 G needle biopsy. Brit J Rad.

